# Liaison haematology in the United Kingdom: Workforce perspectives of workload and labour demand

**DOI:** 10.1111/bjh.70643

**Published:** 2026-06-25

**Authors:** Jonathan Massie, Nicola Ransome, Josh Wright, Sue Pavord, Alison Leary, Adele Stewart‐Lord

**Affiliations:** ^1^ Workforce British Society for Haematology London UK; ^2^ Transfusion Department NHS Blood and Transplant Bristol UK; ^3^ York and Scarborough Teaching Hospitals NHS Foundation Trust Scarborough Hospital Hull UK; ^4^ Haematology Sheffield Teaching Hospitals NHS Foundation Trust Sheffield UK; ^5^ Clinical Haematology Oxford University Hospitals NHS Foundation Trust Oxford UK; ^6^ College of Health and Life Sciences London South Bank University London UK

**Keywords:** haematology, health workforce, liaison haematology

To the Editor,

Unseen labour is a feature of healthcare work and has been studied in the UK National Health Service for approximately 50 years.^1^ Skilled labour provided ‘ad hoc’, such as consultations and advice, is rarely accounted for in workforce planning and often fails to attract organisational support. Many specialties frequently provide advice; however, despite a relatively large demand for haematology advice, little is known about this work and its value within healthcare.^2^, ^3^

These challenges are seen, to a greater or lesser degree, in many areas of healthcare where advice and guidance work is provided. The extent to which the work is studied is variable, but it is better recognised in specialties providing regular advice and guidance such as microbiology and psychiatry where studies have tried to quantify the time commitments and cost‐effectiveness of this work.^4^, ^5^

Internationally a range of terms have been used to describe this work within haematology, including ‘consultative haematology’ and ‘liaison haematology’ (LH).^6^, ^7^ However, the extent to which these terms overlap is poorly described. Existing literature predominantly uses the term ‘consultative haematology’, but this term is not used in the United Kingdom. A recent study led to the formal definition of ‘liaison haematology’ as follows^7^:

*‘Liaison haematology’ is the workload associated with the provision of haematological advice, both clinical and laboratory, as either formal or informal*:

*(a) Advice and guidance, both scheduled and ad‐hoc, via various mediums to other healthcare professionals. This can be outside or within haematology and is in respect of patients who have or are felt to have a problem requiring haematological expertise. These patients may not be under the care of the person providing liaison haematology for the healthcare issue in question*.

*(b) Context to interpretation of laboratory tests*.

Using this formal definition, we investigated the pressures on the LH workforce in the United Kingdom, including demand and barriers to effective service delivery.

A mixed methods approach was taken, beginning with semi‐structured interviews to identify the data that key stakeholders considered most relevant. Following this, elements identified were then measured in a questionnaire among a larger haematology workforce sample.

Key stakeholders were defined as ‘haematology professionals employed as a clinical/service or educational lead’.

An initial recruitment email for the interview phase one, and the questionnaire phase two, was sent out to all British Society for Haematology (BSH) members. The BSH is the primary organisation representing haematologists in the United Kingdom and also has an international membership, with over 3300 members in total.

Participants were asked to return the questionnaire if they were UK‐based and felt that they delivered LH in accordance with the previously published definition.^7^

Limitations of this study include potential selection bias, with participants experiencing large volumes of LH more likely to contribute. While this study is limited to UK participants, findings are relevant to professionals outside the United Kingdom, both in haematology and other frequently consulted specialties.

Ethical approval was obtained from London South Bank University.

The study included 22 key stakeholder interviews and 152 respondents to the questionnaire phase, demographic information provided in Table 1. Participants covered a broad range of centres, both tertiary and secondary, across England and Scotland.

**TABLE 1:** Demographics of interviewees and questionnaire respondents.

Interview participants	Educational leadership role	Clinical leadership role	Clinical and educational leadership roles	Grand total (*n* = 22)
Haematology consultant doctors	2	6	8	16
Consultant clinical scientists	1	1	0	2
Lead biochemical scientists	0	1	1	2
Lead nurse	0	0	1	1
Lead pharmacist	0	1	0	1

Questionnaire respondents	Haematology consultant (*n* = 103)	Haematology registrar (*n* = 25)	Healthcare scientist (*n* = 15)	Other^a^ (*n* = 9)	Grand total (*n* = 152)
Years in current role					
0–9	39	25	6	6	76
10–19	38	0	5	1	44
20–29	21	0	1	2	24
30+	4	0	3	0	7
Do you currently specialise in					
Haemato‐oncology	12	1	2	3	18
Medical (non‐malignant) haematology	37	0	4	3	44
Both	54	24	9	3	90
What type of organisation are you employed with?					
Secondary	60	6	5	2	73
Tertiary	38	18	9	5	70
Other	5	1	1	1	8
Which nation are you based in?					
England	93	22	13	7	135
Northern Ireland	1	1	0	0	2
Scotland	6	1	0	2	9
Wales	3	1	2	0	6

^a^ Other includes registered nurse, pharmacist, other physician.

Interviews were analysed and coded using content analysis, generating 24 separate codes, which were used to develop questions for the subsequent questionnaire. The codes were categorised into three overarching categories. Selected quotations from each category are provided in Table S1.

Interviewees discussed their own LH work, highlighting a mismatch between the actual time spent on LH and its formal recognition in job plans.

Routes to access advice varied; many participants described phone calls for discussion happening unpredictably, while others used shared inboxes or scheduled meetings to reduce interruptions. Interviewees reported formal routes were frequently bypassed, with users preferring to seek advice informally from clinicians they knew and trusted.

A frequently identified issue was the lack of formal recognition for LH as a defined service, which many felt limited investment and strategic oversight. The nature of this study attracted many haematologists who enjoy and take a particular interest in liaison work; however, in interviews they often described a feeling of isolation without a clear ‘liaison haematology community’.

There were suggestions around how to structure regional LH services. Several interviewees touched on this area, proposing two potential models:

- Supporting and developing regional and national networks of ‘liaison haematologists’;
- Formalising the use of regional or national experts to provide advice in their specific subspecialty to a wide range of organisations.

These insights were then investigated further through the questionnaire phase. Respondents reported that LH work arrives from a wide range of modalities; 111 (73%) of respondents reported six or more different routes in use within their organisation. There was significant variability in the ability to record the advice provided (Figure 1A).

**FIGURE 1:**
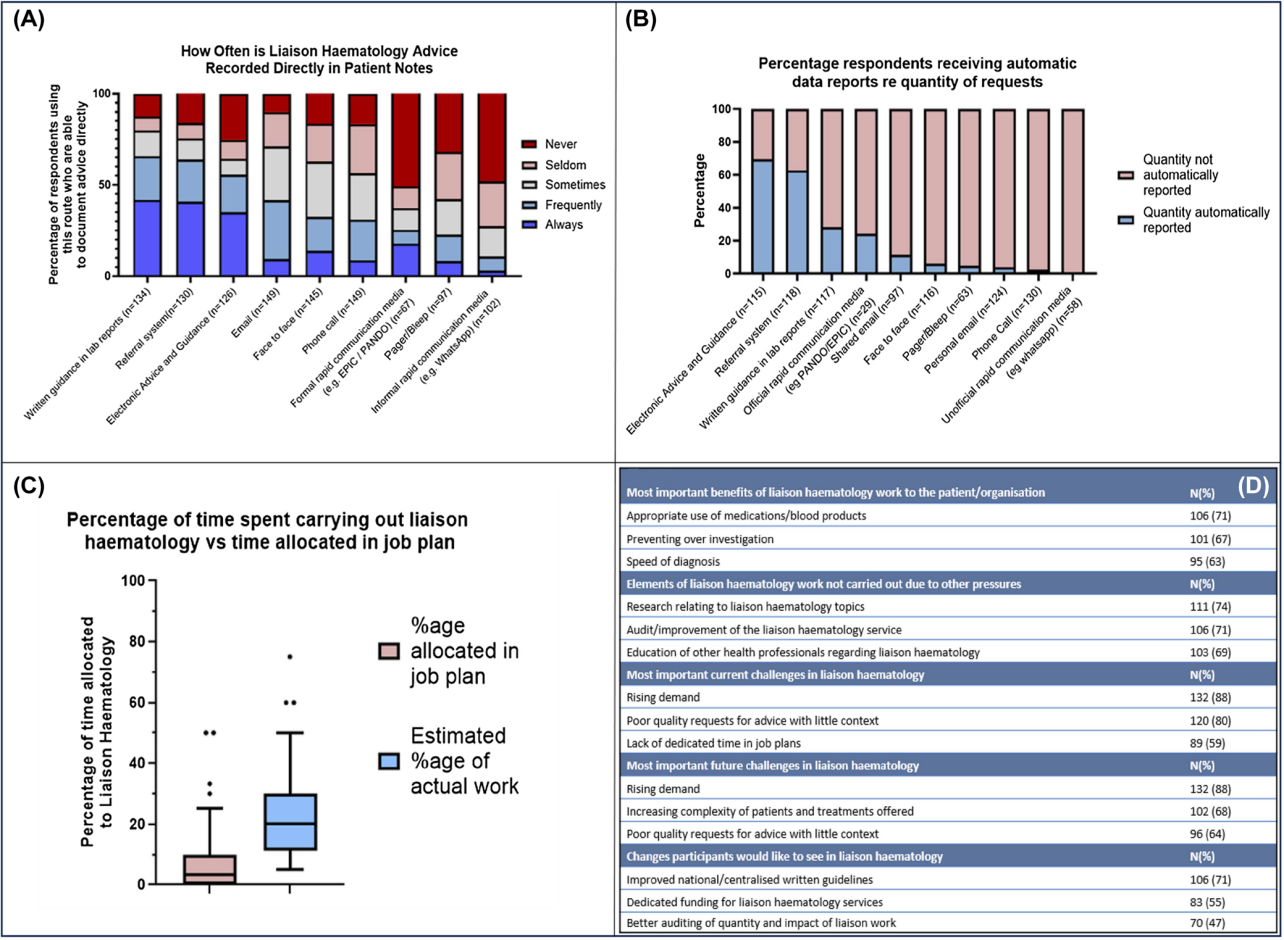
(A) Frequency with which participants estimate they are able to document their advice directly, broken down by the route through which the request arrived. (B) Proportion of participants receiving regular data reports regarding quantity of liaison haematology queries arriving through each route. Percentages are calculated relative to the number of respondents who reported using each route. (C) Tukey plot comparing the proportion of their working week participants estimate they spend carrying out liaison haematology versus the proportion of their time allocated in formal job plans. (D) Codes generated during data analysis from the semi‐structured interviews and how these topics were grouped when designing the phase two questionnaire. LH, liaison haematology.

Work was also frequently carried out for other hospitals; 121 (80%) of respondents reported covering multiple hospital sites including 61 (40%) also providing advice to hospital sites outside of their organisation.

The quantity of requests was sporadically recorded and rarely reported to providers (Figure 1B). In this context, only 8 (5%) respondents reported that data on demand was used to inform workforce planning. Data were also rarely used in determining funding of LH work; only 20 (13%) respondents received funding relative to the number of requests received. Many participants (*n* = 35, 23%) reported no dedicated funding for their LH service.

Most respondents (*n* = 129, 85%) reported having a job plan. Of these, 31 (24%) stated that it did not indicate any time for LH work. Frequent underestimation of the work in job plans was noted (Figure 1C). A majority of respondents (*n* = 102, 68%) also reported undertaking LH work outside of contracted working hours on a daily or weekly basis. Additionally, 110 (73%) reported that LH responsibilities interrupted other work commitments at least weekly.

Participants were asked what areas of the work they wished to devote more time to; this ‘work undone’ was primarily related to LH research (*n* = 111, 74%) or service improvement (*n* = 106, 71%). However, some LH work which might more directly impact care was also affected. Some participants reported that they had insufficient time to adequately respond to advice requests from professionals from other organisations (*n* = 24, 16%) or to reach out proactively to other professionals to advise on abnormal lab results (*n* = 23, 15%).

Participants were asked to identify the most important benefits of LH, as well as the most important challenges facing LH at present, and in the future. Benefits identified by participants were predominantly related to efficient use of resources (Figure 1D). Participants also highlighted the importance of LH in empowering clinicians to manage patients locally (*n* = 85, 57%) and in supporting cases where management or diagnosis was not straightforward (*n* = 81, 54%). Prominent challenges identified related to staffing and formal recognition of the work (Figure 1D). There was also recognition that increasing subspecialisation might lead to problems in future, as haematologists are encouraged to focus on narrower areas of expertise (*n* = 63, 42%).

Participants indicated changes they wished to see in the provision of LH. These focussed on increasing the recognition of LH work, both nationally and within their organisations (Figure 1D). Some participants advocated for recognition of ‘liaison haematology’ as a subspecialism (*n* = 68, 45%).

The findings of this study demonstrate that LH labour demand is a sizable part of participants' workload. LH is poorly represented in job plans and frequently takes place outside of working hours, increasing risk to the service or individual patient and increasing pressure on unseen parts of the workforce. Providers of LH indicate significant organisational benefits such as reducing resource misuse by preventing unnecessary tests and treatment, supporting community care and safer treatment of complex patients.

Documentation of advice, which is critical for safety, accountability, streamlined patient care and auditing purposes, is often not carried out by the person giving the advice. Systems that encourage and facilitate documentation of advice may encourage safer practice. Similarly, responses suggest difficulties in tracking the quantity of advice requests, as data are not always routinely provided. Instead, clinicians report having to count requests manually.

Quantifying the benefits of the work is equally important to justify its funding and drive improvement. Objective measures have been used by other specialties; for example, liaison psychiatry services have shown that timely specialist advice can reduce readmission rates.^8^ Examples also exist within haematology; patient blood management initiatives have measurably improved surgical outcomes and reduced blood usage.^9^

This study highlights several areas in which LH might develop, to improve both efficiency and quality of service. These include facilitating the recording of advice, as well as demand data and potentially tying this to funding and workforce planning.

The data collected in this study reveals a complex and varied picture of LH across the United Kingdom, confirming a requirement for skilled labour that is often unseen, unsupported and unaccounted for. International practice is likely more diverse, but clinicians providing advisory work in other settings may experience similar challenges.

## AUTHOR CONTRIBUTIONS

**Alison Leary:** Conceptualization; funding acquisition; writing – review and editing; data curation. **Jonathan Massie:** Conceptualization; methodology; investigation; validation; writing – original draft; writing – review and editing; visualization; formal analysis; data curation. **Sue Pavord:** Investigation; writing – review and editing; conceptualization; data curation. **Adele Stewart‐Lord:** Conceptualization; investigation; funding acquisition; writing – original draft; methodology; validation; visualization; writing – review and editing; formal analysis; project administration; data curation; supervision; resources. **Josh Wright:** Writing – review and editing; investigation; conceptualization; data curation. **Nicola Ransome:** Conceptualization; investigation; writing – original draft; methodology; validation; visualization; writing – review and editing; formal analysis; data curation.

## FUNDING INFORMATION

This work was funded by the British Society for Haematology.

## CONFLICT OF INTEREST STATEMENT

The authors have no conflicts of interest to disclose.

## Supporting information


**Table S1.** Selected quotes to illustrate interviewee experiences of liaison haematology.

## Data Availability

The data that support the findings of this study are available on request from the corresponding author. The data are not publicly available due to privacy or ethical restrictions.
