# Supplementary material for: Liaison haematology in the United Kingdom: Workforce perspectives of workload and labour demand
Source: Br J Haematol. 2026 Jun 25;209(2):842–6. doi: 10.1111/bjh.70643 (PMC13462209; doi:10.1111/bjh.70643)
Supplement: Supplementary file 1 [file BJH-209-842-s001.docx]

**Supplementary Table 1 - Selected quotes to illustrate interviewee experiences of liaison haematology.**

| Your Liaison Haematology Work | |
| --- | --- |
| Interviewees discussed:  - How their liaison work is distributed within their week, and how their official job plans compare with reality - How their work is recorded - Training in liaison haematology - How the work impacts them psychologically. | *“..... you end up doing more and more and more squeezing things into what becomes a fictional job plan at the end of the day.”* TRANSCRIPT 18 |
|  | *“.....I trust that the receiver of the information is documenting what I said and whether or not that's a good way of trusting the system. I don't know.”* TRANSCRIPT 7 |
|  | *“......because all the focus in terms of CPD and training days, it's never around liaison. It's the unsexy bit of haematology.”* TRANSCRIPT 8 |
|  | *“I think my thrombosis knowledge is reasonable, but if it's something really complicated then I might sort of e-mail my thrombosis, colleague and say, do you mind going in and telling me what to put for this?”* TRANSCRIPT 9 |
| Liaison Haematology Within Your Organisation | |
| Interviewees discussed:  - How liaison haematology work is distributed/delegated within their teams - The structures in place to ensure consistency of advice - What the benefits/outcomes of liaison haematology are - Which areas of liaison haematology work do they feel are left undone due to other pressures | *“.....my medical colleagues [referring to haematology consultants] won't be doing it... they don't want to get involved in an advice and guidance system.”* TRANSCRIPT 18 |
|  | *“We haven't set up specific training programmes or assessments for whether a registrar's competent to give advice in the middle of the night. What we do is we have several hours induction for anyone going to on call Rota.”* TRANSCRIPT 16 |
|  | *"...competency includes them actually doing 100 items as a log and then get checked by senior cancer pharmacists and myself and then they would need to read all the trust and local policy and some regional policies. They would have to shadow a member of staff on day therapy unit handling queries."* TRANSCRIPT 22 |
|  | *“......there's all sorts of things I'd love to do about going out of the community and looking at trying to identify high and low ferritin and early intervention, but we just don't have the time.”* TRANSCRIPT 16 |
| Challenges and Sustainability in Liaison Haematology | |
| Interviewees discussed:  - Challenges currently facing liaison haematology, and future challenges they anticipated - Ideas to improve the sustainability of liaison haematology services | *“.....what I think would be the most useful thing that could come out of any research to do with haematology liaison is having a unified voice.”* TRANSCRIPT 20 |
|  | *“I think at the moment we're basically just firefighting to try and keep our heads above the water.”* TRANSCRIPT 15 |
|  | *“I think we suffer because haematology is poorly taught at med school and people come out of it incredibly confused about haematology and they don't know which blood counts are worrying which ones aren't, and so subsequently we get a lot of referrals that don't need to be sent. So our role is not just to provide information, but to educate as well.”* TRANSCRIPT 6 |
|  | *“I'm fed up of telling new business managers, they change every 18 months... There's no sort of memory in the organisation for what you've been trying to tell them for years.”* TRANSCRIPT 15 |
|  | *“It was an average about 7 minutes for me, but we've agreed for standard advice and guidance, it's not just the haematology, but across all the trust. It's 10 minutes per advice and guidance, and when you add that up, I think it works out to one PA. So as 240 minutes is 24 advice and guidance episodes.”* TRANSCRIPT 16 |
|  | *“I do feel there should be some contracting with organisations. So that there is appropriate reimbursement for that advice that's given to the other trust where we're actually not employed at.”* TRANSCRIPT 21 |
|  | *“a lot of my liaison haematology is done by like, random emails by colleagues I get on really well with. Or a phone call at 5:00 at night from, you know, someone who I had a cup of coffee with two days before. Or walking through ITU and being asked a query. So that's, you know, it's the being out and about and getting to know people.”* TRANSCRIPT 17 |
